# Structural insights into lipid-dependent reversible dimerization of human GLTP

**DOI:** 10.1107/S0907444913000024

**Published:** 2013-03-14

**Authors:** Valeria R. Samygina, Borja Ochoa-Lizarralde, Alexander N. Popov, Aintzane Cabo-Bilbao, Felipe Goni-de-Cerio, Julian G. Molotkovsky, Dinshaw J. Patel, Rhoderick E. Brown, Lucy Malinina

**Affiliations:** a Structural Biology Unit, CIC bioGUNE, Technology Park of Bizkaia, 48160 Derio, Spain; b European Synchrotron Radiation Facility, 38043 Grenoble, France; c Shemyakin–Ovchinnikov Institute of Bioorganic Chemistry, RAS, Moscow 117997, Russian Federation; d Structural Biology Program, Memorial Sloan–Kettering Cancer Center, New York, NY 10021, USA; e Hormel Institute, University of Minnesota, Austin, MN 55912, USA

**Keywords:** glycolipid transfer protein, selectivity, sulfatides, lipid-mediated homodimerization, GLTP fold

## Abstract

Human glycolipid transfer protein (hsGLTP) forms the prototypical GLTP fold and is characterized by a broad transfer selectivity for glycosphingolipids (GSLs). The GLTP mutation D48V near the ‘portal entrance’ of the glycolipid binding site has recently been shown to enhance selectivity for sulfatides (SFs) containing a long acyl chain. Here, nine novel crystal structures of hsGLTP and the SF-selective mutant complexed with short-acyl-chain monoSF and diSF in different crystal forms are reported in order to elucidate the potential functional roles of lipid-mediated homodimerization. In all crystal forms, the hsGLTP–SF complexes displayed homodimeric structures supported by similarly organized intermolecular interactions. The dimerization interface always involved the lipid sphingosine chain, the protein C-terminus (C-end) and α-helices 6 and 2, but the D48V mutant displayed a ‘locked’ dimer conformation compared with the hinge-like flexibility of wild-type dimers. Differences in contact angles, areas and residues at the dimer interfaces in the ‘flexible’ and ‘locked’ dimers revealed a potentially important role of the dimeric structure in the C-end conformation of hsGLTP and in the precise positioning of the key residue of the glycolipid recognition centre, His140. ΔY207 and ΔC-end deletion mutants, in which the C-end is shifted or truncated, showed an almost complete loss of transfer activity. The new structural insights suggest that ligand-dependent reversible dimerization plays a role in the function of human GLTP.

## Abstract 2

It is shown that dimerization is promoted by glycolipid binding to human GLTP. The importance of dimer flexibility in wild-type protein is manifested by point mutation that ‘locks’ the dimer while diversifying ligand/protein adaptations.

## 1. Introduction   

Regulated interaction between specific proteins, commonly referred to as reversible dimerization, serves as a fundamental mechanism by which cells are able to control many key processes (Kuriyan & Eisenberg, 2007 ▶). Examples include normal processes such as dimerization-induced activation of various growth-factor receptors by their ligands as well as pathological events such as those occurring during the dimerization of the transmembrane precursor amyloid protein in Alzheimer’s disease. Accordingly, the control of dimerization at protein–protein contact regions has emerged as a new strategy for drug development (Rollins *et al.*, 2000 ▶).

Many dimerization events take place in biomembranes, involving both transmembrane and monotopic integral proteins and enabling cell-surface binding events to initiate signalling cascades within the cell interior. In contrast, the role of dimerization in controlling the action of amphitropic peripheral proteins that can exist in water-soluble and lipid-bilayer-bound states (Burn, 1988 ▶; Johnson & Cornell, 1999 ▶) is often less clear. Among such proteins are the so-called lipid-transfer proteins, which reside in the cell cytosol but also bind to membranes transiently and reversibly to achieve intermembrane lipid transfer. The potential role of dimerization in regulating the function of lipid-transfer proteins remains largely unexplored, but could involve the regulation of protein translocation on/off the membrane or loading/unloading of lipid cargo while the protein is docked on the membrane surface.

Previously, we determined the crystal structure of human glycolipid transfer protein (hsGLTP) in the lipid-free form and in complex with various glycosphingolipids (GSLs; Malinina *et al.*, 2004 ▶, 2006 ▶; Samygina *et al.*, 2011 ▶). The structure belongs to the all-α class and consists of two layers of helices, which form a sandwich with a hydrophobic pocket for lipid chains referred to as the GLTP fold (Structural Classification of Proteins; http://scop.mrc-lmb.cam.ac.uk/scop). In human GLTP, a glycolipid headgroup recognition centre located on the protein surface enables selective binding of the GSL head and ceramide amide groups *via* multiple specific interactions (Malinina *et al.*, 2004 ▶). Only minor changes are needed for different GSL types (Malinina *et al.*, 2006 ▶; Samygina *et al.*, 2011 ▶), consistent with the broad specificity of GLTP for various GSLs (Brown & Mattjus, 2007 ▶; Abe, 1990 ▶; Kenoth *et al.*, 2010 ▶). Encapsulation of lipid chains within the hydrophobic pocket involves a cleft-like gating mechanism that results in two glycolipid-binding modes. In the ‘sphingosine-in’ mode, encapsulation of both the acyl and sphingosine chains occurs within a single GLTP monomer and has only been observed for GSLs containing *N*-oleoyl acyl chains (Malinina *et al.*, 2004 ▶; Samygina *et al.*, 2011 ▶). In the ‘sphingosine-out’ mode, common to all other (over a dozen tested) GSLs, the sphingosine chain projects outwards and enters the hydrophobic interior of a partner GLTP monomer as part of a lipid-mediated homodimeric arrangement. Other approaches such as analytical centrifugation, gel-filtration and dynamic light scattering (DLS), which all operate in solution and use different ranges of sample concentrations, show different percentages of GLTP dimerization from negligibly small to almost complete (Malinina *et al.*, 2004 ▶; Zhai *et al.*, 2009 ▶; Samygina *et al.*, 2011 ▶), indicating reversibility of the dimer formation. *In silico* interaction propensity analyses of human GLTP (Malinina *et al.*, 2006 ▶) predict strong enhancement of dimer formation when GLTP binds GSL in the sphingosine-out mode. We therefore hypothesize that reversible homodimerization of GLTP–GSL complexes can play an important role in GLTP function.

To elucidate the structural dynamics of the GLTP–GSL dimer and associated adaptations, we comparatively analyzed nine novel crystal structures of wild-type GLTP (wtGLTP) and the sulfatide-specific mutants D48V and A47D/D48V complexed with *N*-lauroyl-containing monosulfatide (*N*-lauroyl-3-*O*-sulfo-galactosylceramide; hereafter referred to as 12:0 monoSF) and disulfatide (*N*-lauroyl-3,6-di-*O*-sulfo-galactosylceramide; hereafter referred to as 12:0 diSF) in different crystal forms and compared them with previously studied GLTP–24:1 monoSF complexes (Samygina *et al.*, 2011 ▶). The resulting structural insights unambiguously indicate a specific hinge-like flexibility of GLTP–SF dimers which is prohibited in the D48V dimers owing to expanded intermonomer contacts. Comprehensive analysis of the dimerization interfaces discloses α-helices 6 and 2 and the protein C-­terminus (C-end) as three specific structural elements that are simultaneously involved in dimer formation and ligand binding by GLTP. The mutations D48V and A47D/D48V, which reversibly regulate access of sphingosine to the hydrophobic pocket (Samygina *et al.*, 2011 ▶), mediate the regulation in the disulfatide (12:0 diSF) differently compared with the monosulfatide (12:0 monoSF) or in the short-acyl-chain sulfatide (12:0 monoSF) compared with the long-acyl-chain sulfatide (24:1 monoSF). In the ‘locked dimer’ of the D48V mutant and the hinge-flexible dimers of wtGLTP, protein–ligand adaptations affect the same regions but operate differently, allowing multiple comparisons and analyses, which are helpful in the search for possible impacts of homodimerization on the function of GLTP. In particular, the important role of the C-­terminus in hsGLTP has become apparent. The C-end is involved in a network of interactions that supports the key residue His140 of the recognition centre in an appropriate conformation for GLTP–GSL recognition. In the D48V mutant, in which the ‘locked hinge’ of the protein–diSF dimer induces significant changes in the position of the C-end upon dimer formation, this network becomes partially disrupted and residue His140 no longer forms the key hydrogen bond anchoring the lipid. Two mutants, ΔY207 and ΔC-end, in which the C-end (Val209) is shifted or truncated show an almost complete loss of transfer activity, confirming the important functional role of the C-terminus in hsGLTP.

## 2. Materials and methods   

### 2.1. Plasmid construction and mutagenesis   

The ORF encoding human GLTP (NCBI GenBank Accession No. AF209704) was subcloned into the pET-30 Xa/LIC expression vector (Novagen) by ligation-independent cloning, enabling cleavage of the N-terminal His_6_-S tag to yield a protein identical in sequence to native GLTP. The site-directed D48V-GLTP mutant was obtained using the QuikChange Site-directed Mutagenesis Kit (Stratagene) and was verified by sequencing. A similar approach was applied to obtain the mutants T207L, ΔC-end and ΔT207 and the double mutant A47D/D48V.

### 2.2. Protein expression and purification   

Transformed *Escherichia coli* BL21 (DE3) cells (Novagen) were grown in Luria–Bertani medium at 310 K, induced with 0.1 m*M* IPTG and grown for an additional 16–20 h at 288 K. Wild-type GLTP and all mutants were purified from soluble lysate by Ni-affinity chromatography as detailed previously (Airenne *et al.*, 2006 ▶). His-tag sequences were removed using factor Xa and the GLTP was further purified by FPLC size-exclusion chromatography using a HiLoad 16/60 Superdex 75 prep-grade column (Amersham Biosciences).

### 2.3. Crystallization and X-ray data collection   

Crystals of wild-type GLTP, D48V-GLTP and A47D/D48V-GLTP complexed with 12:0 monoSF or 12:0 diSF were grown by the hanging-drop vapour-diffusion method using PEG 3350 or 8000 (15–20%) as precipitant and 100 m*M* MES pH 5–7 containing 30–150 m*M* NaCl as buffer (Malinina *et al.*, 2004 ▶, 2006 ▶; Samygina *et al.*, 2011 ▶). All lipids were obtained from Avanti Polar Lipids (Alabaster, AL, USA). Protein–lipid complexes were prepared by mixing protein and lipid in a 1:1 molar ratio using lipids dissolved in ethanol (∼40% EtOH). Crystals were transferred into well solution containing 20% glycerol as cryoprotectant, mounted in a fibre loop and flash-cooled in a cold nitrogen stream. X-ray data were collected at 100 K using synchrotron radiation on beamline ID 23-1 at the ESRF, Grenoble, France, except for crystal form 1 of wtGLTP complexed with 12:0 monoSF and crystal form 2 of wtGLTP complexed with 12:0 diSF, data for which were collected at CIC bioGUNE at the Cu *K*α wavelength (1.54 Å) using an X8 PROTEUM system (Bruker) with a CCD detector. All data were processed and scaled using the *HKL*-2000 program suite.

### 2.4. Structure determination and refinement   

All structures were determined by the molecular-replacement (MR) method using the programs *AMoRe* (Navaza, 1994 ▶) or *MOLREP* (Vagin & Teplyakov, 2010 ▶) with the previously refined complexes as models (Malinina *et al.*, 2004 ▶, 2006 ▶; Samygina *et al.*, 2011 ▶). Refinement was performed with *REFMAC* (Murshudov *et al.*, 2011 ▶), alternating with manual model rebuilding using *TURBO-FRODO* and *Coot*. The *ARP*/*wARP* automatic procedure was used to add solvent molecules (Lamzin & Wilson, 1993 ▶). Final structures were validated by *PROCHECK*. The coordinates have been deposited in the Protein Data Bank. Data-collection and refinement statistics, together with space groups and unit-cell parameters, are summarized in Tables 1 ▶ and 2 ▶.

### 2.5. Fluorescent lipids   

*N*-[(11*E*)-12-(9-Anthryl)-11-dodecenoyl]-1-*O*-β-galactosylsphingosine (AV-GalCer) and 1-acyl-2-[9-(3-perylenoyl)­nonanoyl]-sn-*glycero*-3-phosphocholine (Per-PC) were synthesized as described previously (Molotkovsky & Bergelson, 1982 ▶; Molotkovsky *et al.*, 1991 ▶). 3-*O*-Sulfo-d-­galactosyl-β1–1′-*N*-[(11*E*)-12-(9-anthryl)-11-dodecenoyl]-d-*erythro*-sphingosine (AV-sulfatide) synthesis relied on a similar approach that will be detailed elsewhere. 1-Palmitoyl-2-oleoylphosphatidylcholine (POPC) was purchased from Avanti Polar Lipids. For the chemical structure of AV-GalCer, see Fig. 2(*c*) of Samygina *et al.* (2011 ▶).

### 2.6. Fluorescence lipid transfer between membranes   

Real-time intermembrane transfer rates of fluorescent glycolipids by wt-GLTP and mutants were obtained by Förster resonance energy transfer using a SPEX FluoroMax spectrofluorimeter (Horiba Scientific), with excitation and emission bandpasses of 5 nm and a stirred (∼100 rev min^−1^) and temperature-controlled (298 ± 0.1 K) sample cuvette holder (Samygina *et al.*, 2011 ▶). Both fluorescent lipids were localized initially to the donor vesicles, formed by rapid ethanol injection and comprised of POPC plus 1 mole % AV-glycolipid and 1.5 mole % Per-PC. Minimal emission by AV-glycolipid occurred upon excitation (370 nm) because of resonance energy transfer to nearby Per-PC. Addition of a tenfold excess of sonicated POPC acceptor vesicles produced minimal change in the fluorescence signal because of the very slow spontaneous transfer of lipids with long acyl chains (Mattjus *et al.*, 1999 ▶; Brown, 1992 ▶). Addition of catalytic amounts of GLTP triggered a sudden, exponential increase in AV emission intensity (415 nm) as the protein transported AV-labelled glycolipids from donor vesicles to acceptor vesicles, creating separation from ‘nontransferable’ Per-PC. Addition of detergent after extended incubation provided a measure of the maximum AV intensity achievable by ‘infinite’ separation from Per-PC. Nonlinear regression analyses using *Origin* 7.0 (OriginLab) provide the initial lipid-transfer rate, ν_0_, for the first-order exponential transfer process. Standard deviations were calculated at the 95% confidence interval. *R* ^2^ values for all of the estimates were >0.96.

### 2.7. Preparation of donor and acceptor vesicles   

Donor vesicles composed of POPC (99 mole %), AV-glycolipid (1 mole %) and Per-PC (1 mole %) were prepared by rapid ethanol injection into buffer rapidly stirred in the cuvette at 298 K as described previously (Mattjus *et al.*, 1999 ▶). Prior to injection, all three lipids were mixed together in hexane, dried under nitrogen and then redissolved in ethanol (HPLC grade). Each ethanol injection (5 µl) contained ∼175 pmol of AV-glycolipid. After dilution, the final ethanol concentration was less than 0.2%. The acceptor vesicles were prepared in the following way. POPC was dried onto a glass round-bottom flask *in vacuo* before hydrating in sodium phosphate buffer pH 6.6 at a 50 m*M* concentration and suspending by vortexing. The suspension was probe-sonicated intermittently under nitrogen until opalescent and was then centrifuged for 90 min at 100 000*g* to remove probe particles and multilamellar vesicles. The size of the acceptor-vesicle populations averaged ∼25 nm in diameter. The final acceptor-vesicle concentration used in the FRET lipid-transfer assay was ∼85 µ*M*, which was 10–15-fold higher than the donor concentration.

### 2.8. Dynamic light scattering   

Dynamic light-scattering (DLS) measurements were performed using a DynaPro Titan instrument from Wyatt Technology Corporation (with a laser of ∼830 nm wavelength). Measurements were performed in the protein concentration range 1–4 mg ml^−1^ in 150 m*M* NaCl, 20 m*M* Tris–HCl buffer pH 8.0 at 291 K. Complexes with lipid were prepared immediately prior to measurement using protein:lipid ratios in complexes of 1:1. All buffers were centrifuged at 27 000*g* for 30 min and filtered through 0.2 µm membrane filters (Whatman). Data were collected and analyzed using the *DYNAMICS* software for the DynaPro Titan instrument (Wyatt Technology Corporation).

## 3. Results and discussion   

### 3.1. ‘Universal’ dimeric crystal structure of GLTP–GSL complexes and DLS analyses   

Among the many GLTP–GSL complexes that we have studied previously (Malinina *et al.*, 2004 ▶, 2006 ▶; Samygina *et al.*, 2011 ▶), the only one that displayed a monomeric crystal structure was GLTP complexed with *N*-oleoyl-containing glucosylceramide (18:1 GlcCer). All other complexes involving GSLs with 8:0, 12:0, 18:1, 18:2 or 24:1 acyl chains and lactose, glucose, galactose or 3-*O*-sulfogalactose headgroups formed dimeric crystal structures. Similar dimeric arrangements also were found in the sulfatide-selective mutants D48V and A47D/D48V complexed with *N*-nervonoyl-containing sulfatide (24:1 monoSF; Samygina *et al.*, 2011 ▶). On the other hand, all previous dimeric complexes formed isomorphous crystals. Therefore, two unresolved questions persist: (i) can similar dimeric structures result from different crystal arrangements and (ii) can GLTP–lipid complexes form a dimeric molecular structure in solution?

With the 24:1 monoSF ligand, we previously showed using DLS analyses that binding to wtGLTP or D48V-GLTP promotes dimerization in solution (Samygina *et al.*, 2011 ▶). Similar DLS analyses demonstrate that binding of 12:0 monoSF and 12:0 diSF by wtGLTP or D48V-GLTP also promotes homodimerization (Supplementary Table S1^1^). Thus, our data suggest that glycolipid-induced homodimerization could be functionally important for GLTP.

### 3.2. Towards a structural dynamics study: different crystal forms of the same complexes   

To explore the structural dynamics of GLTP–GSL dimers, we cocrystallized 12:0 monoSF and 12:0 diSF in complex with wtGLTP and the SF-selective mutants D48V and A47D/D48V in different crystal forms. We determined nine novel crystal structures that could be classified into two groups. Table 1 ▶ contains the X-ray data and refinement statistics for class 1 crystals, which are isomorphous to previously studied GLTP crystals (Malinina *et al.*, 2004 ▶, 2006 ▶; Samygina *et al.*, 2011 ▶). Table 2 ▶ shows the X-ray data parameters for the same complexes crystallized in distinct crystal forms. To highlight the general difference in crystal arrangements, Table 2 ▶ includes two additional parameters: *N*, the number of protein monomers in the asymmetric unit, and *V*, the mean volume per protein monomer calculated by dividing the asymmetric unit volume by *N*. In class 1 crystals, *N* is always 1 and *V* equals (53–55) × 10^3^ Å^3^, which corresponds to the crystal content being approximately half water. In class 2 crystals, *N* is 2, 4 or 1, indicating the potential structural distinctions between monomers which are not related by crystallographic symmetry. The value *V* also varies significantly, indicating distinctions between crystal polymorphs. For instance, in crystal form 2 *V* is about 80 × 10^3^ Å^3^, which corresponds to the crystal content being ∼2/3 water.

### 3.3. Do the systematic variations in isomorphous crystals indicate dimer flexibility?   

Despite their isomorphism, class 1 crystals do not possess completely equivalent unit-cell parameters. In contrast, the parameters show systematic variations related to the complex type (Supplementary Table S2). In other words, small reproducible deviations (∼3 Å) of the unit cells distinguish the complexes of neutral GSLs (GalCers and LacCers) from those of sulfatides (12:0 monoSF, 24:1 monoSF or 12:0 diSF) in wild-­type GLTP. Similarly, SF complexes with mutant D48V systematically deviate from those with wtGLTP or the double mutant A47D/D48V. The systematic nature of these variations in different complex types implies that the homodimeric structure of the GLTP complexes possesses intrinsic flexibility.

### 3.4. Similar dimer arrangement in different crystal forms   

Although the crystal packing differs in various crystal forms (Tables 1 ▶ and 2 ▶), we found a similar dimeric structure in all crystals, indicating the non-randomness of GLTP–SF homodimerization. For instance, the dimer formed by two molecules in the asymmetric unit of crystal form 2 is similarly arranged as the dimer observed in the original crystal belonging to space group *C*2 (Malinina *et al.*, 2004 ▶). In crystal form 3, the four molecules comprising the asymmetric unit form two dimers similar to the original *C*2 dimer. Moreover, in crystal form 4, which belongs to the hexagonal space group *P*6_4_22, the GLTP–diSF complex also forms a homodimer with a symmetry-related partner as in the original *C*2 dimer. Importantly, in all of these crystals the homodimeric arrangements are identical, indicating that conserved and specific intermonomer inter­actions occur which regulate and promote this dimerization.

### 3.5. The hinge-like flexibility of wild-type dimers *versus* the locked dimer of D48V-GLTP   

**Figure 1:**
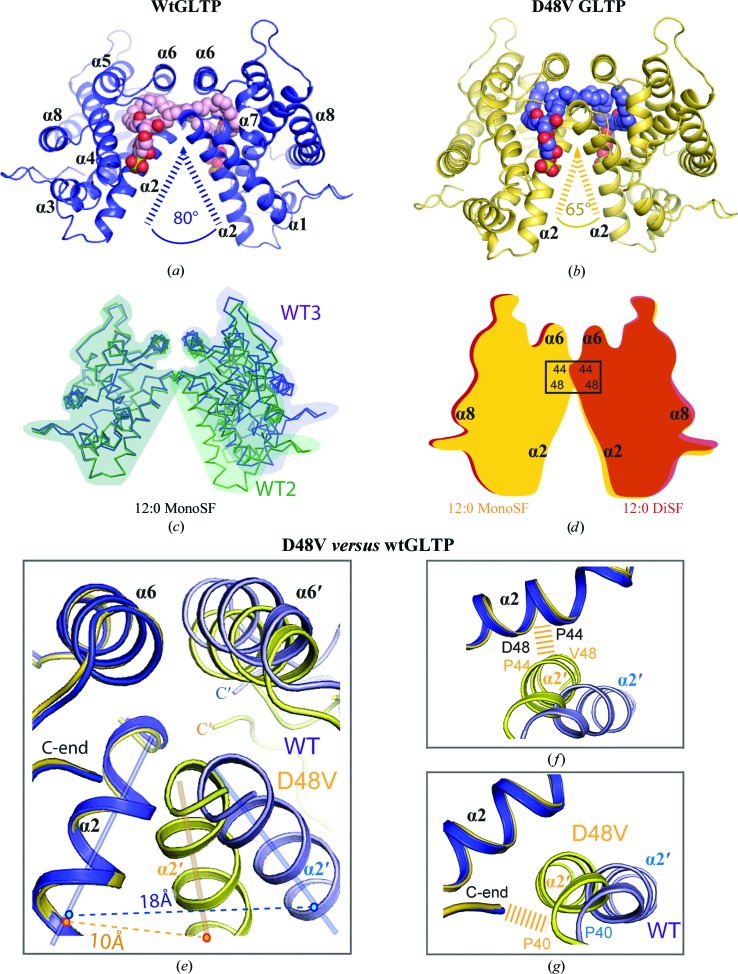
Dimeric arrangement, flexibility and intermolecular contacts in wild-type GLTP (wtGLTP) and the D48V mutant complexed with the short-acyl-chain sulfatide 12:0 monoSF. (*a*, *b*) Open dimer conformation in wtGLTP (*a*) *versus* the locked dimer of D48V (*b*). Protein chains are coloured blue for wtGLTP and gold for the D48V mutant. Sulfatide atoms are coloured red, blue and green for oxygen, nitrogen and sulfur, respectively, while C atoms are coloured pink for the lipid bound to wtGLTP and blue for the lipid bound to the D48V mutant. The eight α-helices of the GLTP fold are labelled α1–α8. The values 80° and 65° indicate the mutual inclination of two α2 helices in the dimeric structure of wtGLTP *versus* D48V-GLTP. (*c*) Comparative superimposition of ‘open’ and ‘closed’ dimer conformations for different wtGLTP crystal forms (referred to as WT3 and WT2). Protein C^α^ chains are coloured blue and green for different dimers. The monomers on the left are superimposed to highlight the range of openness of the monomer on the right for the different dimers. Blue and green semi-transparent solid shapes additionally highlight the superimposition of the left monomers and the range of openness of the right monomer for the different dimers. Ligand molecules are omitted for clarity. (*d*) Schematic highlighting the closed and locked conformation of the D48V dimers. Complexes with 12:0 monoSF and with 12:0-diSF are shown by solid shapes coloured yellow and red–brown, respectively, with α2, α6 and α8 indicating selected helices and the rectangle denoting the hydrophobic core that ‘locks’ the dimer. For the superposition of C^α^ chains, see Supplementary Fig. S1(*c*). (*e*–*g*) Protein–protein′ dimeric contact regions shown by comparative superimposition of wtGLTP and D48V: overall view (*e*) and two zones of expanded contacts in the locked dimer (*f*, *g*). Superpositioning details and colour codes are as in (*a*) and (*c*). Wide dashed bands show expanded contacts. Solid straight lines are conditional axes of α2 helices, while dashed lines with values indicate the midpoint distances between them (*t* distances in Table 3 ▶).

Fig. 1 ▶(*a*) shows the overall dimeric view of wtGLTP complexed with *N*-lauroyl-containing monosulfatide (12:0 monoSF) in sphingosine-out mode. The outwardly extending sphingosine chain enters and interacts with the hydrophobic pocket of the partner GLTP monomer, as well as making antiparallel cross-bridging hydrophobic contacts with the sphingosine chain projecting from the partner GLTP. In addition to sphingosine–sphingosine and sphingosine–protein contacts, another contribution to the monomer–monomer interactions within the dimer is made by the multiple protein–protein contacts analyzed in the next section.

Fig. 1 ▶(*b*) shows an overall view of the D48V mutant complexed with 12:0 monoSF, which shares similarity with the wild-type complex (Fig. 1 ▶ *a*). However, an essential distinction between the two dimers becomes apparent upon careful comparison: the wtGLTP dimeric arrangement is more ‘open’ than that of the mutant. A reasonable measure of ‘dimer openness’ is the angle between the two α2 helices of partner monomers, which is ∼80° in the wild-type complex (Fig. 1 ▶ *a*) and ∼65° in the mutant complex (Fig. 1 ▶ *b*). To elucidate the flexibility of the dimeric structure in different crystals/complexes, we calculated the inter-helical α2–α2′ angle and the midpoint distance between these helices (hereafter referred to as the θ angle and the *t* distance, respectively) for all nine crystal structures and some related GLTP complexes previously deposited in the PDB (Table 3 ▶) using the program *INTERHLX* (K.Yap, University of Toronto). In addition, using the *CCP*4 suite, we estimated the size of each monomer–monomer surface contact area, which is an usual measure of ‘contact strength’ in structural analyses (Janin & Chothia, 1990 ▶; Bahadur & Zacharias, 2008 ▶). Table 3 ▶ summarizes the analyses and reveals several interesting findings. Firstly, the hinge-flexibility of the dimer arrangement is apparent for wtGLTP–SF complexes. Even within the same complex, 12:0-monoSF–wtGLTP, the θ angle and *t* distance vary significantly in the different crystal forms (68.2–80.2° and 15.4–19.5 Å, respectively), reflecting changes in dimer openness. These characteristic features are illustrated in Fig. 1 ▶(*c*), in which two crystal structures of wtGLTP–SF are shown in a conditional superposition: the monomers on the left are superimposed so that the range of openness for the two dimeric forms can be clearly observed in the monomers on the right. To further assess the intrinsic nature of these variations, two dimers belonging to the same crystal (form 3 in Table 3 ▶) were analyzed for changes in dimer openness (Supplementary Fig. S1*a*). The observed differences are comparable with those found in different complex types belonging to isomorphous crystals (Supplementary Fig. S1*d*). The inherent differences in dimer openness within wtGLTP–SF complexes are most dramatically illustrated by superimposition of the most open and most closed dimers (Supplementary Fig. S1*b*).

It is noteworthy that the monomer–monomer surface contact areas (Table 3 ▶) of various wtGLTP–SF dimers, as well as the contribution of the protein–protein contacts, display quite small deviations from their mean values (∼1850 and ∼1050 Å^2^, respectively), indicating conserved dimer stability over a wide range of openness. The first noticeable deviations in the surface contact areas occur in wtGLTP–disulfatide complexes, which probably indicate a slightly different mutual orientation of monomers compared with wtGLTP–monosulfatide complexes. In any case, our analyses support the following general conclusions: (i) the dimeric conformation of the wtGLTP–SF complex exhibits hinge-like flexibility and (ii) the dimer stability (as measured by the values of the surface contact area) is practically the same for all dimer conformations (open and closed) in wtGLTP–SF complexes.

In contrast, Fig. 1 ▶(*d*) compares the homodimeric arrangement of the D48V mutant complexed with either monoSF or diSF. Only ‘closed’ conformations characterized by a very narrow range of θ angles (62.6–65.5°) are observed in different complexes/crystals (Table 3 ▶). Although these values are similar to those for closed dimer conformations of wtGLTP complexes (differing by only ∼3–5°), the *t* distances are noticeably smaller in the mutant (∼10.5 *versus* 13.6–15.4 Å; Table 3 ▶), indicating that D48V mutation promotes a shift of each monomer towards the other. This shift is emphasized schematically in Fig. 1 ▶(*d*) by the contacts involving Pro44 and Val48 in the D48V dimers, while the conservation of the closed conformation of D48V dimers in different crystal complexes can be clearly seen in their superposition (Supplementary Fig. S1*c*). The extra monomer–monomer interactions arising in the mutant and described in detail in the next section stabilize the closed conformation of D48V–SF dimers, which we therefore refer to as a ‘locked dimer’.

### 3.6. Intermolecular contacts supporting the homodimeric structure   

A magnified view of the protein contact area in the dimers is shown in Fig. 1 ▶(*e*) by comparative superimposition of wtGLTP and D48V-GLTP complexed with 12:0 monoSF, with one monomer superimposed and with the sulfatide molecule omitted for clarity. In general, the intermolecular dimeric interactions involve multiple protein–protein, lipid–lipid and protein–lipid contacts between two partners, with the absolute majority of these contacts being hydrophobic. Protein–protein interactions (Fig. 1 ▶ *e*) include (i) multiple van der Waals contacts between two α6 helices that face each other in the dimer, (ii) interactions between the two mutually inclined α2 helices and (iii–iv) contacts between the tip of the α2 helix and the α6 helix as well as the C-terminus of the partner molecule. Since the partner is rotated and shifted in the D48V mutant compared with that of wtGLTP (Fig. 1 ▶ *e*), the dimeric contacts differ in the complexes. To analyze the differences, we have grouped the intermolecular dimeric contacts in Table 4 ▶ by their contacting structural elements. In Table 4 ▶, each pair of interacting elements is characterized by their contacting amino-acid residues and the total count of short interatomic separations (less than 4 Å), which we refer to as interatomic contacts. The last parameter is used for comparative purposes because of its sensitivity to change in the dimer arrangement. In this context, a value of 0 indicates much weaker interaction rather than no interaction at all.

#### 3.6.1. wtGLTP dimer   

Examination of Table 4 ▶ reveals that the α6–α6′ contact is the main protein–protein interaction in the wild-type dimer. This interaction includes residues Trp142, Ile143, Lys146, Ile147, Ala150, Ala154 and Tyr153 from one protein monomer and counterpart residues, listed in reverse order, from the partner monomer. The α2 helix tip contacts the C-end′ of the partner molecule through Pro40 and Val209*, whereas Val41 (next to Pro40) contacts the α6′ helix through Ala151 (not listed in Table 4 ▶) as well as the α6′ helix of the partner monomer through Val144* (Table 4 ▶). The α2 and α2′ helices undergo only very limited van der Waals interaction through Pro44 and Pro44*. It is noteworthy that the last observation is true for all complexes of wtGLTP.

The second significant contributor to dimer intermolecular interactions arises from the lipid–lipid′ hydrophobic contacts and multiple lipid–protein′ contacts, the majority of which are a consequence of the sphingosine-out binding mode. The contribution of lipid–protein′ interactions to dimer stabilization is greater than any other (40 contacts in addition to 25 of α6–α6′ type; Table 4 ▶), consistent with our DLS-based observation indicating that lipid binding promotes protein dimerization.

#### 3.6.2. Dimer of the D48V mutant   

Compared with the wild-type protein, some interfacial regions in the D48V dimer contain noticeably expanded intermolecular contacts. The first region involves the α2–α2′ hydrophobic core formed by residues Pro44, Ala47 and Val48 with their counterparts, as was originally found in the D48V mutant complexed with 24:1 monoSF in the sphingosine-in binding mode (Samygina *et al.*, 2011 ▶). With 12:0 monoSF, the mutant uses a similar contact region for the major protein–protein′ interaction in the dimer (25 *versus* 16 α6–α6′ contacts; Table 4 ▶). The specific changes in the α2′ position of the D48V dimer are shown in Fig. 1 ▶(*f*).

The second expanded interaction region arises from contributions from the C-end and the tip of the α2′-helix of the partner protein (Fig. 1 ▶ *g*). Importantly, the expansion strengthens the C-end–α2′ interaction compared with the wild-type dimer (18 contacts in the mutant *versus* two contacts in wtGLTP; Table 4 ▶) and affects the function of GLTP, as demonstrated in §[Sec sec3.11]3.11. In turn, the lipid–lipid′ and the lipid–protein′ interactions become weaker in the dimer of the D48V mutant (see Table 4 ▶) as indicated by the reduced number of interatomic close contacts, which are reminiscent of those encountered in the sphingosine-in binding mode of 24:1 monoSF complexed with the same mutant (Samygina *et al.*, 2011 ▶).

### 3.7. Binding mode of 12:0 monoSF with wtGLTP *versus* the D48V mutant   

**Figure 2:**
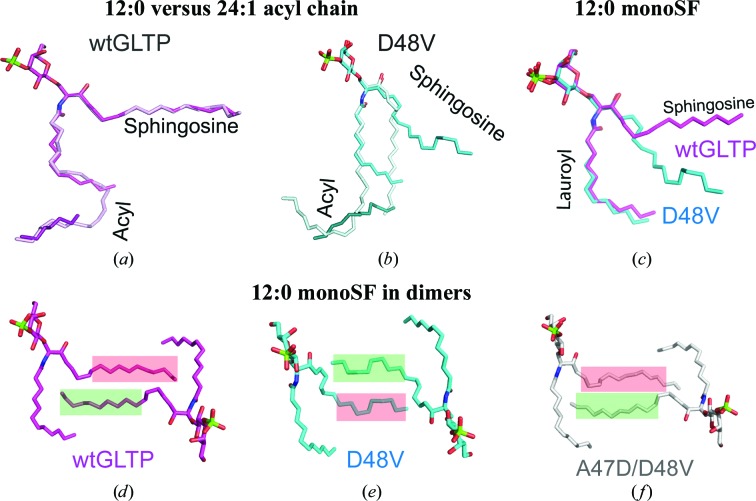
Various conformations of 12:0-monoSF in wtGLTP and GLTP mutants. (*a*, *b*) Comparative superpositions of 12:0 monoSF with 24:1 monoSF as bound to wtGLTP (*a*) *versus* D48V (*b*). Protein moieties are omitted after being used for superimpositioning. Sulfatide atoms are coloured red, blue and green for oxygen, nitrogen and sulfur, respectively. 12:0-monoSF C atoms and extraneous hydrocarbons found in the hydrophobic pocket are coloured magenta in wtGLTP and cyan in D48V; 24:1 monoSF C atoms are coloured light magenta and light cyan, respectively. (*c*) Comparative superposition of 12:0 monoSF conformations in wtGLTP and D48V. Colour codes are as in (*a*) and (*b*). (*d*–*f*) Dimeric arrangements of the sphingosine-out modes in wtGLTP (*d*), D48V (*e*) and A47D/D48V (*f*). Colour codes are as in (*a*) and (*b*), except for the C atoms of A47D/D48V, which are shown in white. Red and green semitransparent rectangles highlight the sphingosine chains of the left and right monomers, respectively.

We previously showed that the D48V mutation switched the binding mode of 24:1 monoSF from sphingosine-out in the wild-type protein to sphingosine-in in the mutant, with noticeably modified lipid conformation (Samygina *et al.*, 2011 ▶). The switching effect was induced by dimer interfacial changes involving the α2–α2′ hydrophobic core (Samygina *et al.*, 2011 ▶). Since the same core becomes the major protein–protein′ contact in the D48V dimer complexed with 12:0 monoSF, we were surprised to discover the sphingosine chain projecting outwards and entering the partner hydrophobic pocket in the sphingosine-out binding mode. The lipid-chain conformations of 12:0 monoSF within wild-type GLTP and the D48V mutant are shown in Figs. 2 ▶(*a*) and 2 ▶(*b*) in comparison with the sphingosine-out and sphingosine-in conformations observed for complexes with 24:1 monoSF. The similarity of the sphingosine-chain arrangements in both of the wtGLTP–SF complexes highlights the difference between two lipid-binding modes in the mutant.

Although the sphingosine-out mode of 12:0 monoSF in the D48V mutant is obvious, careful comparison (Fig. 2 ▶ *c*) shows that it is a modified sphingosine-out conformation that is adapted to the closed dimer conformation of the mutant. Because the double mutation A47D/D48V restores the open dimer conformation along with the local negative charge repulsion in the dimer (Samygina *et al.*, 2011 ▶), we also studied the crystal structure of the double mutant A47D/D48V complexed with 12:0 monoSF.

The analyses of the dimeric arrangements of 12:0 monoSF within wtGLTP (Fig. 2 ▶ *d*), the D48V mutant (Fig. 2 ▶ *e*) and the double mutant A47D/D48V (Fig. 2 ▶ *f*) revealed differences in their sphingosine-out conformations. In wtGLTP (Fig. 2 ▶ *d*), the sphingosine chain (red rectangle) passes above the partner chain (green rectangle). In D48V-GLTP (Fig. 2 ▶ *e*), the positioning of the sphingosine chains is reversed. However, in the double mutant A47D/D48V the original lipid-chain arrangement found in wtGLTP is restored (Fig. 2 ▶ *f*). Importantly, the sphingosine-out mode promotes increased sphingosine–sphingosine′ cross-bridging contacts in the dimeric structure of wtGLTP complexes (six close contacts; Table 4 ▶). In contrast, in the D48V modified sphingosine-out mode (Fig. 2 ▶ *e*) this interaction is much weaker (three close contacts; Table 4 ▶) and comparable (no close contact) with the sphingosine-in mode observed for 24:1 monoSF within the same mutant (Supplementary Table S3). The lipid–protein′ interactions even more closely resemble those of the sphingosine-in mode (18 close contacts *versus* 16, respectively; Table S3). Therefore, both dimers (sphingosine-in-bound 24:1-monoSF and sphingosine-out-bound 12:0-monoSF) have very similar monomer–monomer′ surface contact areas (1580 *versus* 1540 Å^2^, respectively; Table 3 ▶) that are noticeably diminished compared with wtGLTP complexes (1800–1890 Å^2^). The diminished monomer–monomer′ contact areas of the D48V–SF dimers imply reduced stabilities, which decrease despite the expanded protein–protein′ contacts (1300–1310 *versus* 1020–1090 Å^2^ in wtGLTP), again emphasizing the importance of the bound ligand for GLTP dimerization.

### 3.8. Interactions of disulfatide with the GLTP recognition centre   

**Figure 3:**
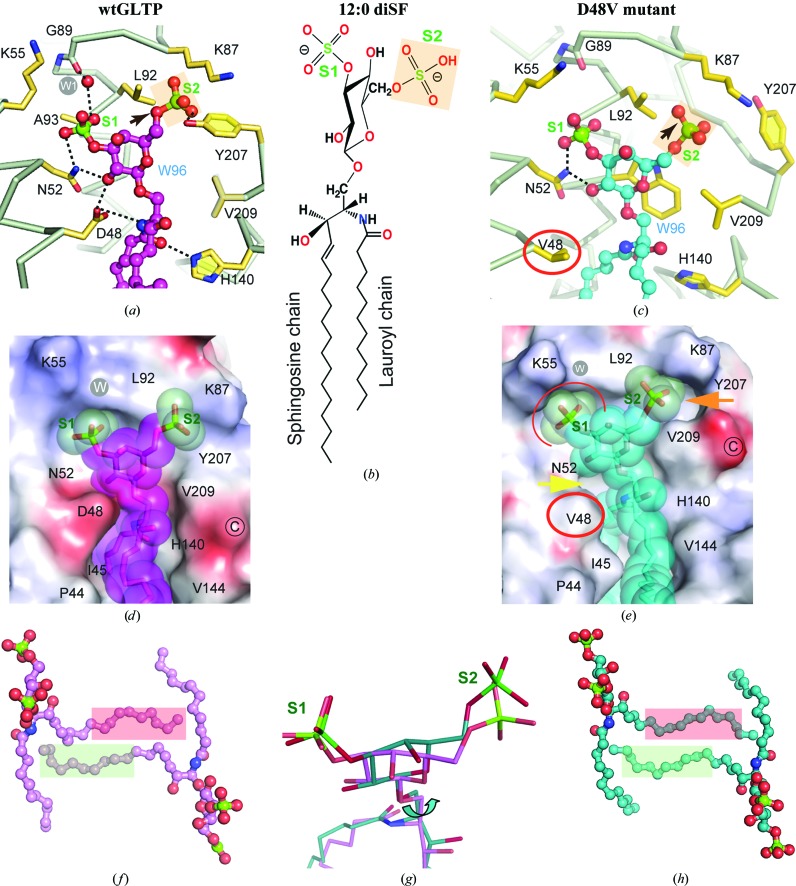
Disulfatide binding to wtGLTP and the D48V mutant. (*a*) 3,6-*O*-Disulfo-Gal headgroup in the wtGLTP recognition centre. Dashed lines indicate hydrogen bonds. Disulfatide atoms are coloured red, blue, green and magenta for oxygen, nitrogen, sulfur and carbon, respectively. Protein C^α^ backbone and side-chain C atoms are coloured silver and gold, respectively. The grey circle labelled W1 or W indicates the conserved water molecule. S1 and S2 (pink rectangles) are 3-*O*- and 6-*O*-sulfo groups, respectively. (*b*) Chemical structure of *N*-lauroyl-3,6-*O*-disulfo-galactosylceramide. (*c*) 3,6-*O*-Disulfo-Gal headgroup in the recognition centre of the D48V mutant. Colour codes and designations are as in (*a*), except for ligand C atoms, which are coloured cyan. The mutated residue 48 is shown in a red circle; the black arrow points out the different conformation of S2 compared with that in wtGLTP (*a*). (*d*, *e*) Electrostatic surface view (blue, positive; red, negative; grey, neutral) of the GLTP recognition centre in the wild-type protein (*d*) and the D48V mutant (*e*) occupied by a disulfatide molecule shown in stick representation within a space-filled semitransparent shape with green-coloured sulfo groups. Colour codes are as in (*a*) and (*b*). The mutated residue is shown in a red circle; arrows point out the ‘empty’ space (filled by water molecules) resulting from the D48V mutation and the conformational change of the S2 group promoting the movement of the C-end. (*f*, *h*) Dimeric arrangements of 12:0-diSF in wtGLTP (*f*) and D48V (*h*), with the ligand in ball-and-stick representation. Colour codes are as in Figs. 2 ▶(*d*) and (*e*). (*g*) Superimposed disulfatide molecules as bound to D48V (cyan) *versus* wtGLTP (magenta).

Fig. 3 ▶ shows the interactions of GLTP with bound *N*-­lauroyl-3,6-*O*-disulfo-galactosylceramide (12:0 diSF). Compared with 12:0 monoSF, disulfatide has an additional sulfo group (the 6-*O*-sulfo group referred to as S2 in Fig. 3 ▶ *b*) located on the opposite side of galactose from the 3-*O*-sulfo group. The network of hydrogen-bond interactions (Fig. 3 ▶ *a*) and van der Waals contacts (Fig. 3 ▶ *d*) between 12:0 diSF and the headgroup recognition centre of wild-type GLTP is similar to that found for monosulfatides (Samygina *et al.*, 2011 ▶), except for sulfo group S2, which is embedded into a recess in the protein surface between residues Leu92 and Lys87 *via* van der Waals contacts. In addition, the 6-*O*-sulfo group forms a hydrogen bond to the OH group of Tyr207 (Fig. 3 ▶ *a*). Disulfatide and all amino-acid residues of the GLTP recognition centre are clearly visible in the electron-density map (Supplementary Figs. S2*a* and S2*e*). The protein structure displays no disturbance compared with wtGLTP complexed with monosulfatides. Hence, we can assume that the disulfatide found in the malaria parasite *Plasmodium falciparum* (Landoni *et al.*, 2007 ▶) can also be bound by human GLTP.

Compared with the wild-type protein, the D48V mutant displays conformational rearrangements of the protein C-­end and partial loss of hydrogen bonding to disulfatide (Figs. 3 ▶ *c* and 3 ▶ *d*). In particular, the mutant lacks both hydrogen bonds to the side chain of residue Asp48, which is now replaced by Val, and also lacks the hydrogen bond to His140. The latter finding was unexpected because this hydrogen bond is considered to be a key interaction in the GLTP recognition centre, since the H140L mutation almost completely inactivates GLTP (Malinina *et al.*, 2004 ▶). This complex is the first example of the ceramide amide group not being hydrogen bonded to the recognition centre of GLTP (Fig. 3 ▶ *c*).

### 3.9. Disulfatide-binding modes within wtGLTP and the D48V mutant   

Interestingly, disulfatide binds to both wtGLTP and the D48 mutant in the sphingosine-out mode (Figs. 3 ▶ *f* and 3 ▶ *h*). This finding indicates small (but important) differences in the mutual positioning of the two monomers in the protein–diSF dimer (compared with the protein–monoSF dimer), which probably result from the additional negative charge in disulfatide. Indeed, careful comparison reveals some monomer–monomer′ contact redistribution in the dimer in the region of the α6–α6′ and α2–α2′ protein interactions and lipid–protein′ contacts (Supplementary Table S3). The result for the D48V dimer complexed with disulfatide is a conserved and more stable sphingosine-out binding mode (Fig. 3 ▶ *h*) compared with the monosulfatide complex as judged by the significantly larger surface contact area (2300–2250 *versus* 1540–1580 Å^2^; Table 3 ▶). Thus, three different adaptations of sulfatide are observed upon binding to D48V-GLTP compared with wtGLTP: (i) a switch from the sphingosine-out mode to the sphingosine-in mode in 24:1 monoSF, (ii) modulation of the sphingosine-out mode in 12:0 monoSF and (iii) conservation of unmodified sphingosine-out conformation in 12:0 diSF, which noticeably increases the monomer–monomer′ surface contact area in the dimer, mainly because of amplification of the sphingosine–protein′ contact (Supplementary Table S3).

### 3.10. Disulfatide–protein adaptability and dynamics   

**Figure 4:**
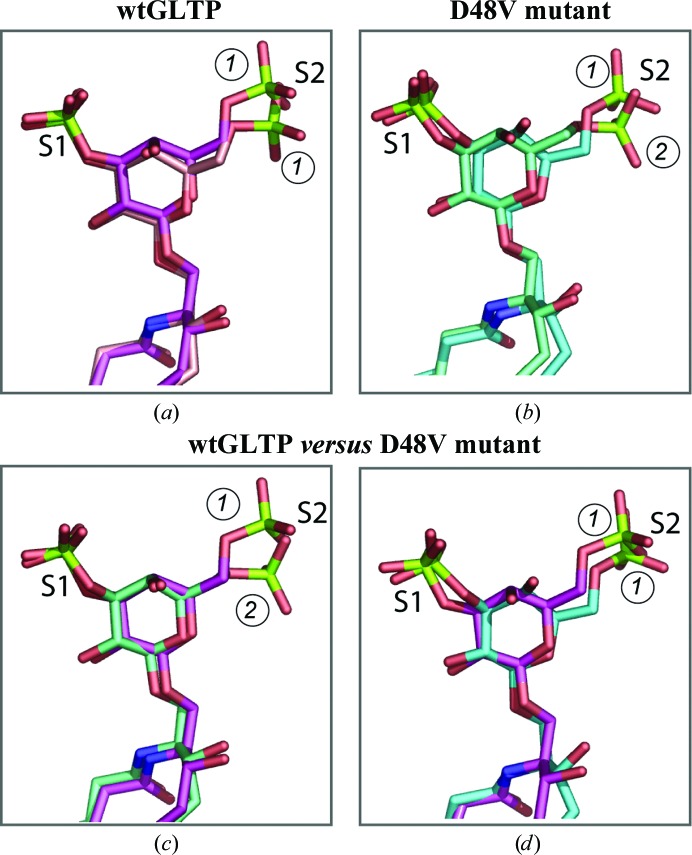
Conformational adaptability of disulfatide headgroups. (*a*) Superimposed ligand conformations in open and closed dimers (crystal forms 1 and 2, respectively) of wtGLTP. Protein moieties were superposed and skipped. Disulfatide molecules are shown in stick representation; colour codes for O, N and S atoms are red, blue and green, respectively. C atoms are coloured magenta for crystal form 1 and pink for crystal form 2. (*b*) Disulfatide conformations in two crystal forms (1 and 4; Tables 1 ▶ and 2 ▶) of D48V. C atoms are coloured cyan for crystal form 1 and light cyan for crystal form 4. Colour codes for other atoms are as in (*a*). (*c*, *d*) Disulfatide in the open dimer of wtGLTP *versus* the two conformations of disulfatide shown in (*b*).

To elucidate the dynamics of intermolecular interactions in the GLTP–disulfatide complex, we comparatively analyzed two crystal forms of wtGLTP and two crystal forms of the D48V mutant complexed with 12:0 diSF. The crystals of the wtGLTP–disulfatide complex represent ‘open’ and ‘closed’ conformations of the dimeric arrangement. In contrast, both D48V–diSF crystals display a closed (‘locked’) dimer conformation but belong to different space groups. In Fig. 4 ▶, the disulfatide conformations are shown by pairwise superpositions, as they are bound to wtGLTP and D48V-GLTP in different crystals, with S1 and S2 indicating the 3-­*O*-sulfo and 6-*O*-sulfo groups, respectively. Two conformational states of the 6-*O*-sulfo group can be distinguished by their different rotational angle around the C5′—C6′ bond and are referred to as conformations 1 and 2 in Fig. 4 ▶. When complexed with wtGLTP (Fig. 4 ▶ *a*), disulfatide adopts conformation 1 in both crystals. However, adaptations within the open and closed dimers necessitate slightly different overall lipid conformations. In D48V-GLTP complexes (Fig. 4 ▶ *b*) disulfatide displays both conformation 1 and conformation 2, demonstrating a different adaptation strategy. However, the overall disulfatide conformation changes less when the 6-*O*-sulfo group switches to conformation 2 (Fig. 4 ▶ *c*) than when conformation 1 is conserved (Fig. 4 ▶ *d*). Thus, conformation 2 appears to be a preferred adaptation for disulfatide within the locked dimer of D48V.

Superpositions of lipids enables identification of those lipid parts which become less flexible upon binding to GLTP (Fig. 4 ▶). For instance, the 3-*O*-sulfo group is similarly positioned in all cases (Fig. 4 ▶) because of specific anchoring by GLTP. In contrast, the 6-*O*-sulfo group adopts different conformations/positions that depend on the protein (wild type or D48V) or the crystal type. Fig. 4 ▶ shows that the ligand edge comprised by the 3-*O*-sulfo group and acyl lipid chain is more firmly anchored to GLTP than the other edge that involves 6-*­O*-sulfo group.

### 3.11. Important role of the C-end in GLTP–lipid binding and homodimerization   

**Figure 5:**
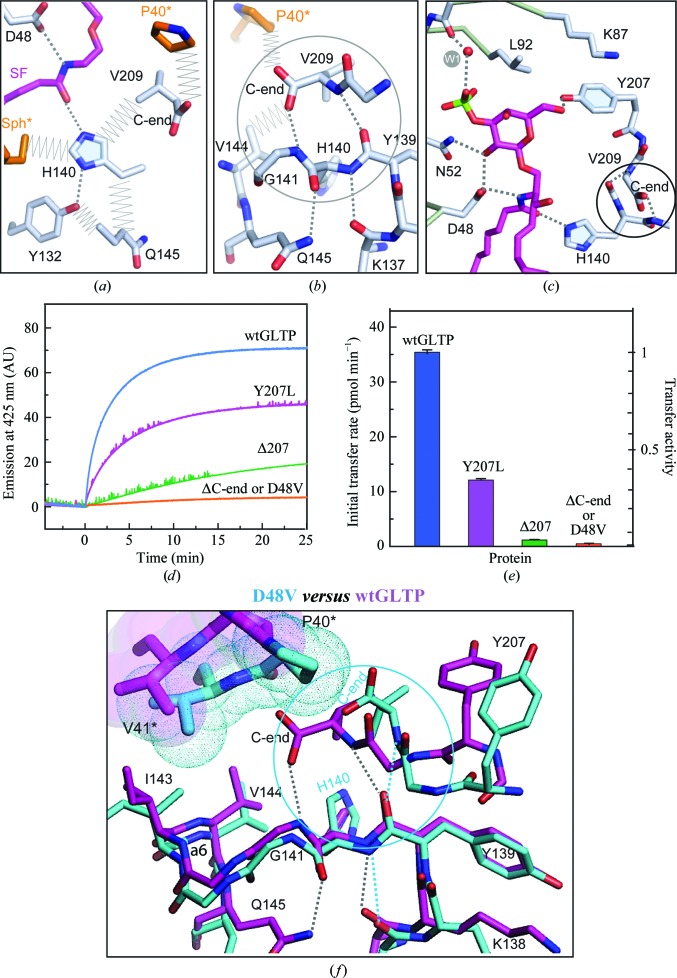
Involvement of the protein C-end in the network of interactions supporting residue His140 in human GLTP. (*a*) The His140 side chain makes a key hydrogen bond to the ceramide amide group and multiple contacts with the environment. Ligand and amino acids are shown in stick representation. C atoms are coloured magenta in the lipid, silver in the amino-acid residues surrounding His140 and orange in the partner monomer. Colour codes for O and N atoms are red and blue, respectively. Dashed lines indicate hydrogen bonds, while zigzags are close van der Waals contacts. The C-terminal residue Val209 simultaneously contacts His140 and Pro40* from the partner. (*b*) The main chain of residue His140 supported by the network of hydrogen bonds involving the C-end of the protein main chain. Designations are as in (*a*). The circled part of the network highlights the C-end contribution. (*c*) Disposition of the circled part of the network in proximity to the recognition centre of hsGLTP (the Val209 side chain is skipped). Colour codes are as in (*a*). (*d*, *e*) Transfer activity assays for wtGLTP and mutants. (*d*) Transfer of the AV-glycolipid by wt-GLTP (blue), Y207L (magenta), Δ207 (green) or D48V/ΔC-end (orange) as a function of time. The increase in fluorescence emission at 415 nm (AV emission) occurs because of decreased Förster resonance energy transfer when AV-glycolipid is removed from donor vesicles containing 3-perylenoyl PC and is transported to POPC acceptor vesicles (see §[Sec sec2]2 for details). The AV signal change does not occur in the absence of POPC acceptor vesicles. (*e*) Transfer activity of GLTP by D48V, Y207L mutations or Y207/C-end deletions. The initial rates of AV-glycolipid departure from the donor vesicles are shown. (*f*) Expanded view of interactions shown in (*b*) for the open dimeric arrangement of wtGLTP complexed with monoSF (magenta) *versus* the locked dimer of the D48V mutant complexed with diSF (cyan). Parts of the partner protein monomer contacting the C-end are highlighted by additional sphere representations in an appropriate colour.

Although His140, one of the most important residues of the GLTP recognition centre, resides on a loop (between helices α5 and α6), the position and conformation of His140 are strictly fixed. The side chain of His140 is strongly supported by a network of interactions with the environment (Fig. 5 ▶ *a*). In the dimer, His140 is surrounded on all sides, with each of its atoms contacting a different residue. Among the contacts are (i) the key hydrogen bond to the ceramide amide group, (ii) a hydrogen bond to the OH group of the conserved residue Tyr132 and (iii–v) van der Waals contacts with the partner sphingosine chain, the side chain of Gln145 and the C-terminal residue Val209. In addition to the side chain, the main chain of His140 also forms a network of hydrogen bonds with the main chain of Lys137 and the C-terminal main chain as well as with the side-chain amide group of Gln145 (Fig. 5 ▶ *b*). The entire network of interactions helps to optimally fix the position of His140 for recognition of the GSL ceramide amide group (Fig. 5 ▶ *c*). Because Val209 makes extensive interactions with His140 and contacts Pro40* of the partner monomer, we hypothesized that dimeric contacts involving Val209 and nearby C-terminal residues reflect changes in conformation that could be important for GLTP function.

Our structural findings shed light on the deleterious nature of deleting the ten C-end residues of GLTP on transfer activity, which is comparable with the effect of the D48V mutation (Figs. 5 ▶ *d* and 5 ▶ *e*). In fact, truncation of the C-­terminus destroys the interaction network with the bound lipid and can be expected to weaken the dimerization potential. The net effect is disturbance of the key hydrogen bond to the ceramide amide group, similar to the effect of the mutation D48V located at the opposite side of the same group (Samygina *et al.*, 2011 ▶). Also, shifting the C-terminal residues by the deletion of Tyr207 (ΔY207) strongly diminished the GLTP-transfer activity. In contrast, the point mutation Y207L, which only disrupts the hydrogen bond between the OH group of Tyr207 and the lipid head (Fig. 5 ▶ *c*), remains ∼50% active (Figs. 5 ▶ *d* and 5 ▶ *e*).

The important role of the C-end in maintaining His140 is further supported by comparative structural analysis of the D48V–diSF complex with wtGLTP complexes (Fig. 5 ▶ *f*). As explained above, compared with wtGLTP dimers, all dimers of D48V mutant are locked. The 6-*O*-sulfo group of disulfatide cannot occupy its position in D48V-GLTP without pushing out Tyr207 (Fig. 3 ▶ *c*). Superposition of D48V–diSF and the wtGLTP complex (Fig. 5 ▶ *f*) shows synchronized pushing of the C-end by Pro40* (along with outward movement of Tyr207) inducing conformational rearrangements of the C-­terminus and partially disrupting the network of interactions supporting His140. In response, His140 changes conformation (Fig. 5 ▶ *f*) and can no longer form the key hydrogen bond to the ceramide (Fig. 3 ▶ *c*).

## 4. Concluding discussion   

In the present study, our new crystal structures reveal lipid-dependent reversible dimerization of human GLTP that could be important for protein function. The key finding is a highly conserved dimeric contact interface in different crystal polymorphs and complexes. The interface encompasses the GSL-binding site and overlaps the putative membrane-docking region for GLTP monomers (Malinina *et al.*, 2006 ▶; Kamlekar *et al.*, 2010 ▶). The finding implies that ligand-mediated reversible homodimerization could be involved in regulating the GLTP–membrane interactions and reducing the exposure of the hydrophobic membrane-interaction region of GLTP upon translocation to the aqueous milieu. By investigating different crystal polymorphs of hsGLTP–SF complexes and the SF-­specific D48V mutant, we comprehensively analyzed the dimer-interface flexibility and the GLTP lipid adaptability.

Our early structural studies of wtGLTP first revealed lipid-mediated protein homodimerization, but its significance was unclear because analytical centrifugation failed to detect GLTP dimerization in solution at low protein concentrations (Malinina *et al.*, 2006 ▶). The recent revelation of dimer formation in D48V complexed with sulfatide containing the 24:1 acyl chain found by X-ray crystallography and by dynamic light scattering (DLS) in solution (Samygina *et al.*, 2011 ▶) indicated the need for further study.

In the present investigation, we again used sulfatide, but varied the chemical structure to elucidate how acyl-chain shortening and/or 6-*O*-sulfo group addition to 3-*O*-sulfo-GalCer regulates the structural adaptations in the highly flexible dimer of wtGLTP and the locked dimer of D48V. Short-acyl-chain sulfatides, 12:0 monoSF and 12:0 diSF, adopt the sphingosine-out mode (Figs. 2 ▶ *a*, 2 ▶ *d* and 3 ▶ *f*) upon binding to the wild-type protein in similar manner as previously shown for wtGLTP complexed with the long-acyl-chain sulfatide 24:1 monoSF (Samygina *et al.*, 2011 ▶). In contrast, the same lipids bound to D48V reveal a diversity of binding modes: (i) the sphingosine-in mode with 24:1 monoSF (Fig. 2 ▶ *b*), (ii) the reversed sphingosine-out mode with 12:0 monoSF (Figs. 2 ▶ *b* and 2 ▶ *e*) and (iii) the sphingosine-out conformation of 12:0 diSF (Fig. 3 ▶ *h*) characterized by redistribution of the intermonomeric contacts (Supplementary Table S3) that noticeably increases the monomer–monomer′ contact area compared with that in the wtGLTP complex. The diversity of the lipid-binding modes in D48V showing the possible lipid-chain adaptations that can occur provides essential insights into the locked dimeric arrangement. Interestingly, ligand adaptations also emerge with wtGLTP complexes bound with disulfatide. Compared with the open dimer, the closed dimeric form promotes rearrangements of the 6-*O*-sulfo group of disulfatide (Fig. 4 ▶ *a*), albeit smaller in magnitude than those that occur in the D48V locked dimer (Figs. 4 ▶ *c* and 4 ▶ *d*). When bound to D48V, the 6-*O*-sulfo group of disulfatide displays two conformational states distinguished by rotation around the C5′—C6′ bond (Fig. 4 ▶ *b*). In fact, this complex revealed not only ligand adaptations but also adaptations of the protein region in proximity to the 6-*O*-sulfo group (Fig. 5 ▶ *f*) and involvement of the C-terminus.

The involvement of the C-terminus in the network of interactions that holds the key residue, His140, of the recognition centre in an appropriate conformation for hsGLTP–GSL recognition (Figs. 5 ▶ *a*–5*c*) is intriguing compared with fungal and algal GLTPs. In the GLTP-like proteins from the alga *Galdieria sulphuraria* (PDB entry 2q52; Levin *et al.*, 2007 ▶) and the fungus *Podospora anserina* (PDB entry 3kv0; Kenoth *et al.*, 2010 ▶), the C-end seems to be involved in supporting other important parts of the GLTP fold. We suggest, therefore, that the C-end plays a unique role in each case and is an important contributor to the functional diversity of the GLTP folds. In human GLTP, the C-terminus serves as an indicator of the openness of the homodimeric structure while also being indirectly involved in supporting the ceramide amide group of the bound GSL.

The new structural insights into the ligand-dependent reversible dimerization of human GLTP changes our understanding of GLTP functionality and suggests that control of dimerization contact regions might provide a new strategy for targeting GSL-mediated pathologies.

## Supplementary Material

PDB reference: GLTP, wild type, complex with 12:0 monoSF, 4h2z

PDB reference: 4gjq

PDB reference: 4gxg

PDB reference: complex with 12:0 diSF, 4gix

PDB reference: 4ghs

PDB reference: D48V mutant, complex with 12:0 monoSF, 4gh0

PDB reference: complex with 12:0 diSF, 4gxd

PDB reference: 4gvt

PDB reference: A47D/D48V mutant, complex with 12:0 monoSF, 4ghp

Supporting information file. DOI: 10.1107/S0907444913000024/lv5031sup1.pdf

## Figures and Tables

**Table 1:** X-ray data-collection and refinement statistics for ‘isomorphous’ *C*2 crystals of wild-type GLTP and GLTP mutants complexed with 12:0 monoSF (monosulfatide) and 12:0 diSF (disulfatide) Values in parentheses are for the highest resolution shell.

Ligand	12:0 monoSF (3-*O*-sulfo-galactosylceramide)			12:0 diSF (3,6-*O*-sulfo-galactosylceramide)	
Protein	wtGLTP	D48V	A47D/D48V	wtGLTP	D48V
Data collection					
Space group	*C*2				
Molecules in the asymmetric unit	1				
Volume per molecule (^3^)	54000				
Unit-cell parameters					
*a* ()	74.60	77.99	76.33	74.77	78.25
*b* ()	49.99	47.24	49.12	50.16	47.32
*c* ()	65.46	62.25	68.12	65.85	63.89
= ()	90	90	90	90	90
()	118.62	125.50	123.58	118.82	126.07
Resolution ()	501.45 (1.481.45)	501.35 (1.401.35)	501.83 (1.861.83)	501.80 (1.861.80)	502.10 (2.122.10)
*R*_sym_ or *R*_merge_ (%)	5.4 (44.9)	7.9 (46.7)	6.9 (44.9)	4.8 (36.8)	10.2 (45.9)
*I*/(*I*)	24.5 (3.12)	17.9 (2.68)	10.0 (2.06)	15.1 (3.2)	12.9 (8.4)
Completeness (%)	98.97 (97.02)	99.6 (99.8)	99.4 (99.9)	97.7 (96.2)	100.0 (100.0)
Multiplicity	7.8 (6.7)	3.9 (2.7)	4.2 (3.4)	3.1 (3.0)	6.8 (6.9)
Refinement					
Resolution ()	151.45	151.35	151.90	151.80	152.10
No. of reflections	35346	40513	15715	10128	10588
*R*_work_/*R*_free_ (%)	15.8/19.0	15.2/18.0	21.3/24.6	18.7/25.0	19.3/22.8
No. of atoms					
Protein	1683	1714	1629	1697	1636
Lipid	49	49	49	53	53
Water/hydrocarbon	238/14	397/16	31/6	160/0	56/12
*B* factors (^2^)					
Protein	27.99	24.54	44.85	31.79	35.30
Lipid	35.47	32.9	48.0	41.78	44.80
Water/hydrocarbon	36.7/44.1	34.8/47.0	44.5	36.64	47.08/57.0
R.m.s. deviations					
Bond lengths ()	0.018	0.015	0.018	0.014	0.018
Bond angles ()	1.74	1.56	1.46	1.38	1.69
PDB entry	4h2z	4gh0	4ghp	4gix	4gxd

**Table 2:** X-ray data-collection and refinement statistics for new crystal forms of wild-type GLTP and the D48V mutant complexed with 12:0 monoSF (monosulfatide) and 12:0 diSF (disulfatide) Values in parentheses are for the highest resolution shell.

Ligand	12:0 monoSF (3-*O*-sulfo-galactosylceramide)		12:0 diSF (3,6-*O*-sulfo-galactosylceramide)	
Crystal	wtGLTP form 2	wtGLTP form 3	wtGLTP form 2	D48V-GLTP form 4
Data collection				
Space group	*P*2_1_2_1_2_1_	*P*2_1_2_1_2_1_	*P*2_1_2_1_2_1_	*P*6_4_22
Unit-cell parameters				
*a* ()	50.96	65.21	50.95	111.11
*b* ()	95.48	84.66	94.77	111.11
*c* ()	132.4	171.88	130.8	75.29
= ()	90.0	90.0	90.0	90.0
()	90.0	90.0	90.0	120.0
No. of molecules in asymmetric unit *N*	2	4	2	1
Volume per molecule *V* (^3^)	80500	59300	79000	67000
Resolution ()	202.0 (2.052.00)	502.40 (2.602.40)	503.20 (3.303.20)	502.95 (3.002.95)
*R*_sym_ or *R*_merge_ (%)	7.5 (63.8)	11.8 (60.0)	10.8 (36.2)	(38.8)
*I*/(*I*)	10.8 (2.0)	13.1 (4.9)	10.5 (4.5)	22.3 (6.5)
Completeness (%)	98.3 (99.56)	97.8 (89.8)	97.9 (97.9)	96.8 (95.6)
Multiplicity	3.9 (3.4)	4.1 (3.9)	5.5 (5.3)	11.9 (12.0)
Refinement				
Resolution ()	152.00	152.40	153.20	152.95
No. of reflections	41480	35197	10128	5885
*R*_work_/*R*_free_ (%)	21.5/23.6	20.9/27.1	18.6/26.3	19.8/28.2
No. of atoms				
Protein	3234	6730	3268	1660
Lipid	98	188	92	53
Water/hydrocarbon	269/13	246/	/30	
*B* factors (^2^)				
Protein	42.8	37.13	17.34	66.48
Lipid	43.2	47.33	22.5	67.2
Water/hydrocarbon	43.63/46.2	32.96	/40.6	
R.m.s. deviations				
Bond lengths ()	0.019	0.013	0.014	0.018
Bond angles ()	1.43	1.53	1.69	1.71
PDB entry	4gjq	4gxg	4ghs	4gvt

**Table 3:** Dimeric structure characteristics in different crystals of GLTPsulfatide complexes

	Mutual 22 inclination and midpoint distance†		Monomermonomer contact surface area‡ (^2^)	
Sulfatide name§ (crystal form)	angle¶ ()	*t* distance¶ ()	Proteinprotein	Total
wt-GLTP				
12:0 monoSF (1)	76.1	18.2	1050	1870
12:0 monoSF (2)	68.2	15.4	1080	1830
12:0 monoSF (3), chains *A*/*B*	77.9	18.1	1090	1890
12:0 monoSF (3), chains *D*/*E*	80.2	19.5	1030	1800
24:1 monoSF (1) (3rzn)	77.2	18.2	1020	1830
24:1 GalCer (1) (2euk ††)	75.2	16.7	1040	1870
12:0 diSF (1)	75.8	18.4	960	1770
12:0 diSF (2)	69.0	13.6	1120	2080
D48V-mutant GLTP				
12:0 monoSF (1)	62.6	10.4	1300	1580
24:1 monoSF (1) (3s0i)	64.8	10.8	1310	1540
12:0 diSF (1)	65.5	10.7	1250	2300
12:0 diSF (4)	63.1	10.8	1260	2250
A47D/D48V double-mutant GLTP				
12:0 monoSF (1)	74.4	15.6	970	1810
24:1 monoSF (1) (3ric)	75.3	15.7	1040	1890

† The line connecting the C atoms of residues 41 and 63 was taken as the 2 helix axis.

‡ Surface-contact areas were calculated with the *CCP*4 suite.

§ Names as cited in the text.

¶ Interhelical angles ( angles) and midpoint distances (*t* distances) were calculated using the *INTERHLX* program (K. Yap, University of Toronto).

†† Nonsulfated ligand from Malinina *et al.* (2006 ▶).

**Table 4:** Intermolecular dimer contacts in wtGLTP and the D48V mutant complexed with 12:0 monoSF, crystal form 1 Structure elements and residues involved in dimeric contacts are given. Only close contacts are considered, with a contact distance of 4.

	wt	D48V
66		
Trp142*	Tyr153	Tyr153
Ile143*	Ala150, Tyr153, Ala154	Ala150, Tyr153
Lys146*	Ala150	
Ile147*	Ile147, Ala150	
Ala150*	Ile143, Lys146, Ile147	lle143
Ala154*	Ile143	
Tyr153*	Trp142, Ile143	Trp142, Ile143
Contacts	25	16
22		
Pro44*		Pro44, Val48
Ala47*		Ala47, Val48
Val48*		Pro44, Ala47
Contacts	0	26
(Tip of 2)6		
Val41*	Val144	Ile143, Val144
Contacts	2	4
(Tip of 2)C-end		
Pro40*	Val209	Val209
Contacts	2	18
Lipidlipid		
Contacts	6	3
Lipidprotein		
Lipid	Val41, Ile45, His140, Ile147, Phe148, Ala151	Pro40, Thr43, Pro44, Ile147
Contacts	40	18

## Footnotes

1 Supplementary material has been deposited in the IUCr electronic archive (Reference: LV5031). Services for accessing this material are described at the back of the journal.
