# Supplementary material for: Structural insights into lipid-dependent reversible dimerization of human GLTP
Source: Acta Crystallogr D Biol Crystallogr. 2013 Mar 14;69(Pt 4):603–16. doi: 10.1107/S0907444913000024 (PMC3606038; doi:10.1107/S0907444913000024)
Supplement: Supplementary file 1 [file d-69-00603-sup1.pdf]

Structural insights into lipid-dependent reversible dimerization of human GLTP

Valeriya Samygina[a], Borja Ochoa-Lizarralde[a], Alexander Popov[b], Aintzane Cabo-Bilbao[a], Felipe Goni-de-Cerio[a], Dinshaw J. Patel[c], Rhoderick E. Brown[d]* and Lucy Malinina[a]*

[a]Structural Biology Unit, CICbioGUNE, Derio, Technology Park of Bizkaia, 48160, Spain, [b]European Synchrotron Radiation Facility, Grenoble, 38043, France, [c]Structural Biology Program, Memorial Sloan-Kettering Cancer Center, New Yoork, NY, 10021, United States, and [d]Homel Institute, University of Minnesota, Austin, MN, 55912, United States

Correspondence email: reb@umn.edu; lucy@cicbiogune.es

Supplementary Material

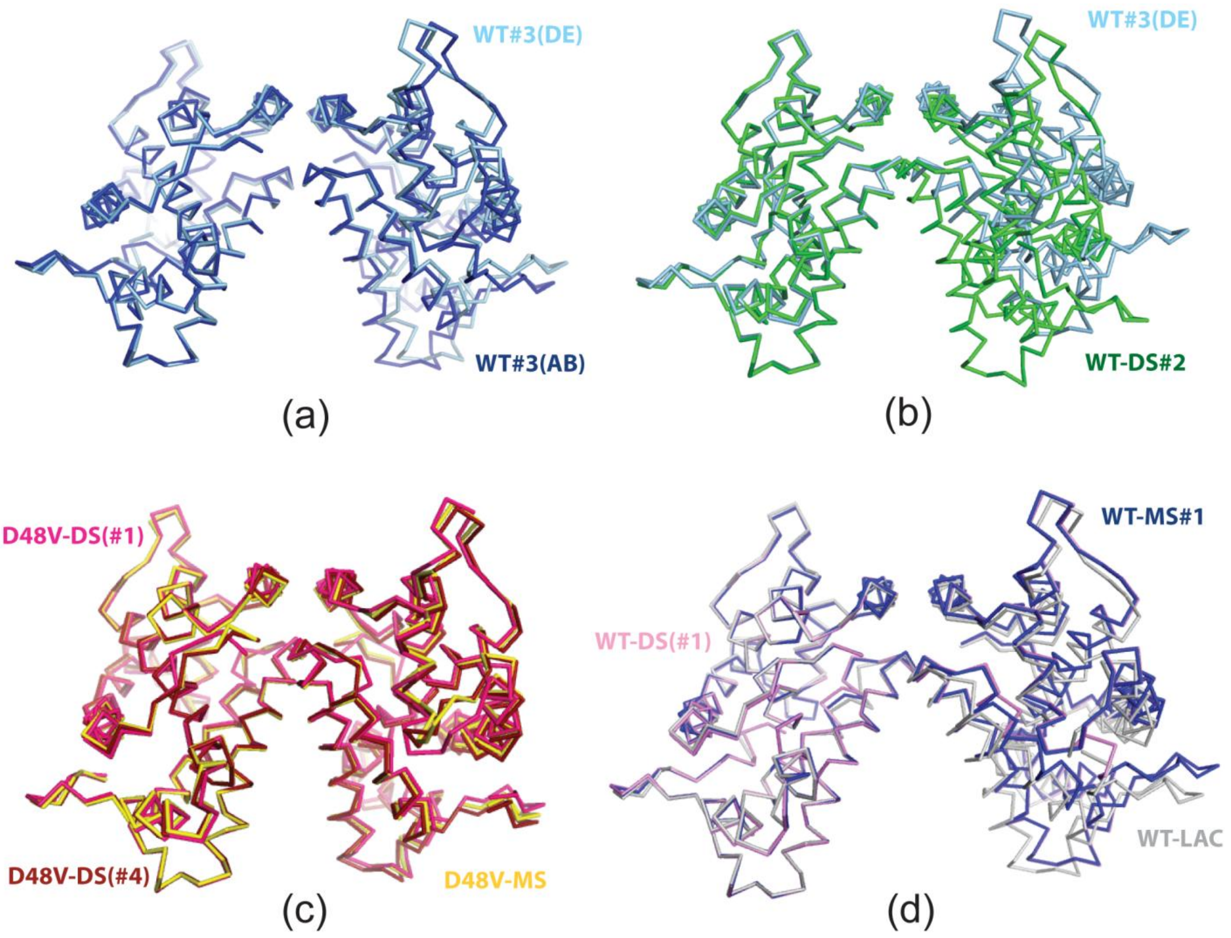

Figure S1

Supplementary Figure S1 Comparative superpositions of GLTP dimers observed in various complexes and diverse crystal forms. Only 'left' monomers are superimposed to highlight the positional distinction (if any) between 'right' monomers because of the different dimer 'openness'. Protein molecules are in Cα-backbone representation; ligand molecules are omitted for clarity. (a) Two dimers, composed of chains A&B (blue), and D&E (light blue), observed in the *same crystal* (form#3) of wtGLTP complexed with 12:0 monoSF. (b) The *most different* (by dimer-openness) among the wtGLTP•SF complexes dimer D&E from (a), shown in light blue, and crystal form #2 of wtGLTP•diSF, shown in green. (c) *Locked dimers* of D48V-GLTP•12:0-monoSF (yellow-colored), and D48V-GLTP•12:0-diSF as in crystal form#1(orange-colored) and crystal form#4 (red-colored). (d) Dimers from isomorphous crystal forms #1 of wtGLTP bound with *sulfated* ligands *versus non-sulfated* one: 12:0 monoSF (blue) and diSF (pink) versus 12:0 LacCer (grey).

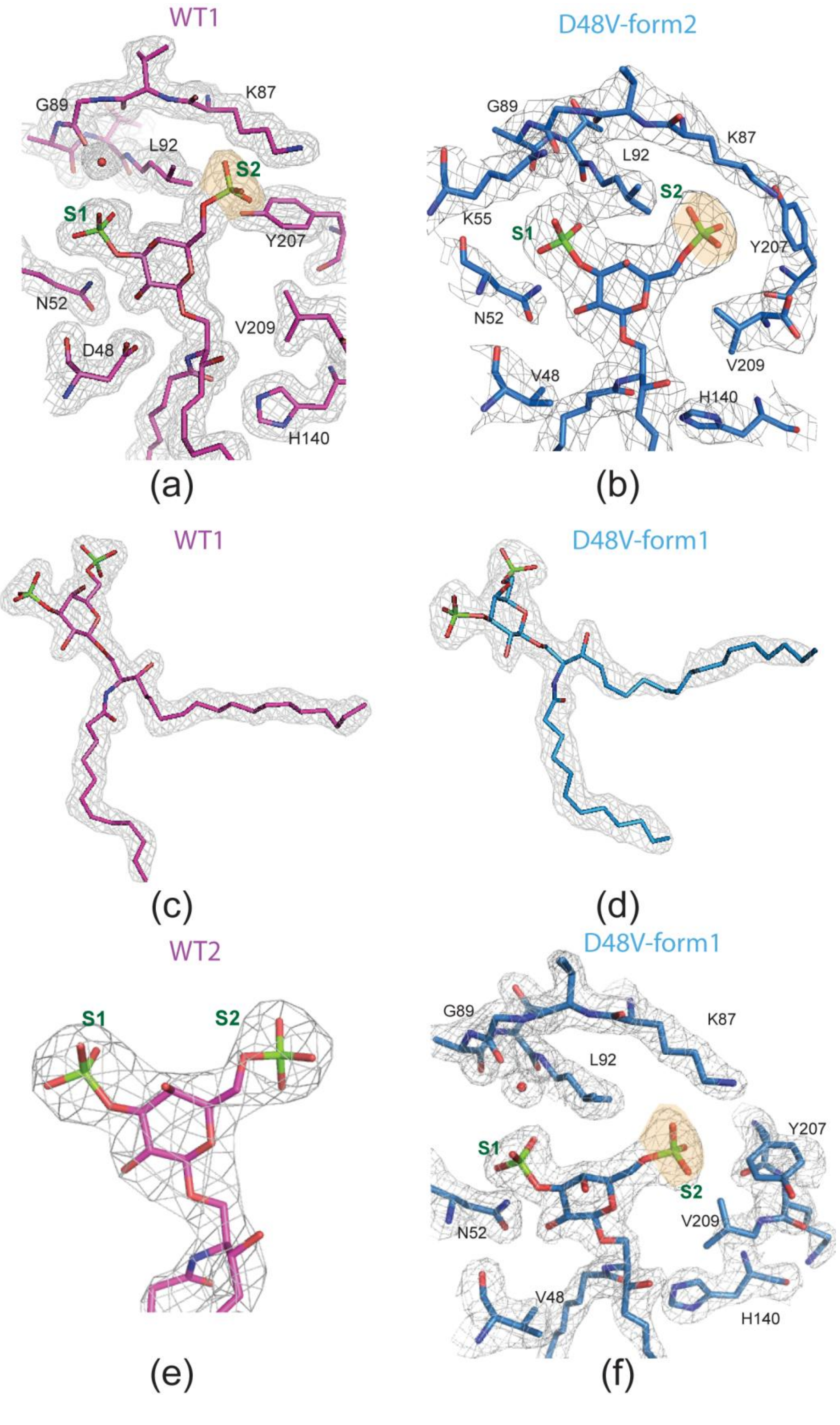
WT1
G89
K87
L92
S2
S1
Y207
N52
V209
D48
H140
(a)
D48V-form2
G89
K87
L92
S2
K55
S1
Y207
N52
V209
V48
H140
(b)
WT1
(c)
D48V-form1
(d)
WT2
S1
S2
(e)
D48V-form1
G89
K87
L92
Y207
S1
S2
N52
V209
V48
H140
(f)

Figure S2

Supplementary Figure S2 Representative 2Fo-Fc electron density maps for different crystal forms of 12:0-diSF bound to wtGLTP (magenta-colored) versus D48V-GLTP (cyan-colored). (a) and (b) Ligand headgroup surrounded by residues of the recognition center in wtGLTP, crystal form#1 (a) and D48V-GLTP, crystal form #4 (b). (c) and (d) Disulfatides within electron density map bound to wtGLTP, crystal form #1 (c) and D48V-GLTP, crystal form #1 (d). (e) Headgroup of disulfatide molecule from the complex with wtGLTP within the low-resolution electron density map, crystal form #2. (f) Same complex as in(b) in crystal form #1. Oxygen and nitrogen atoms are red and blue, respectively. Maps are countered at 1 sigma level.

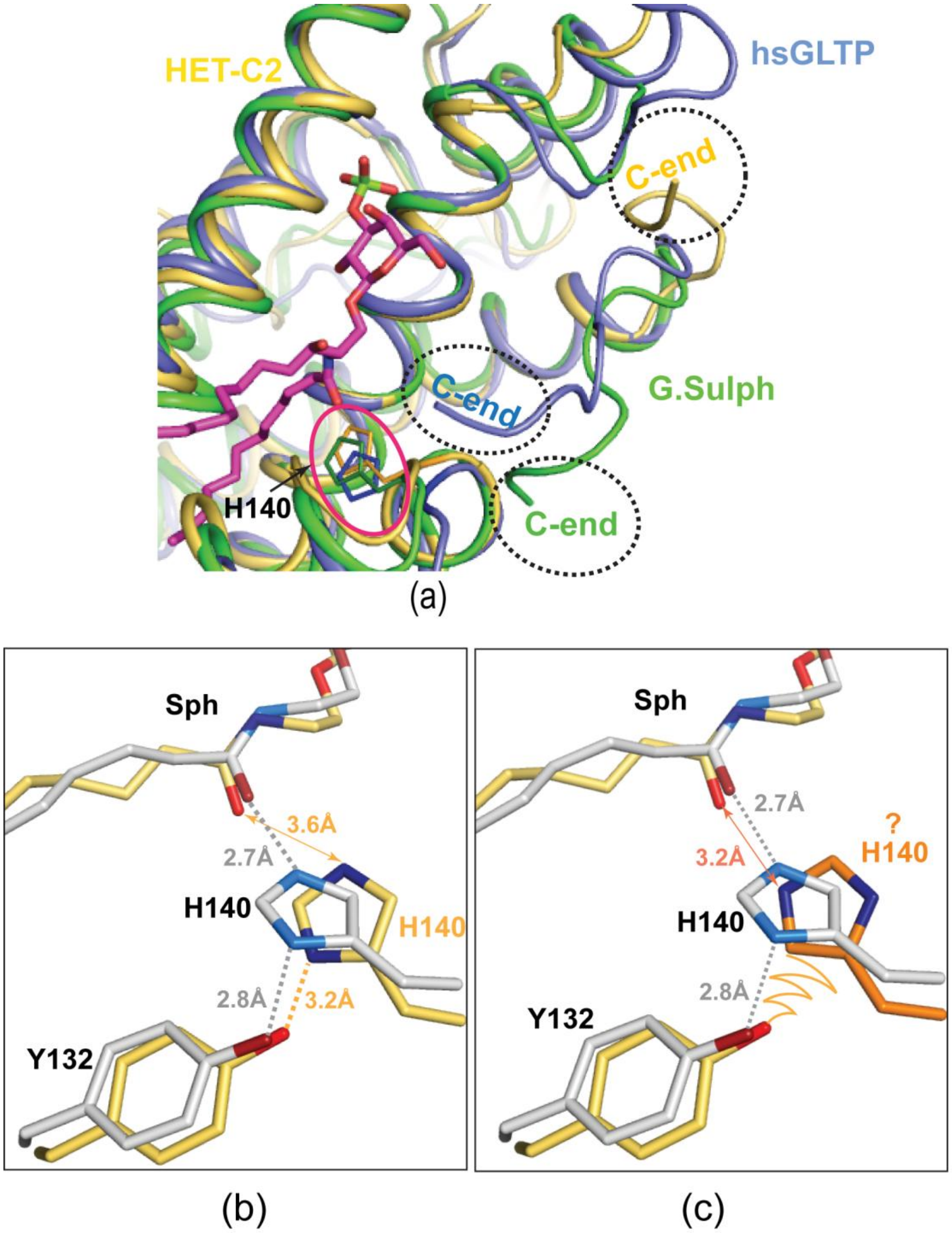

Figure S3

Supplementary Figure S3 C-end positions in GLTP-like proteins and His140 ‘knockdown’ paralleled with C-end outward movement, in locked-dimer of mutated human GLTP complexed with diSF. (a) Different positions of C-ends in hsGLTP, HET-C2 from fungi and GLTP-like protein from *G. Sulphuraria*. Protein chains are in ribbon representation, lipid (12:0-monoSF as bound to hsGLTP) is in stick representation. Color codes are blue, yellow and green for hsGLTP, HET-C2 (PDB 3kv0) and *G. Sulphuraria* (PDB 2q52) colored green. C-ends are highlighted by black

dashed ovals. Side chain of H140 in hsGLTP and counterparts from other proteins are highlighted by magenta oval. (b) and (c) Shift of residue H140 found in the locked-dimer of D48V•diSF compared to H140-position observed in all other complexes. Independently on the rotational angle ((b) or (c)) around Cβ-Cγ bond (that cannot be discriminated by the electron density map), the changed position of H140 worsens its ligand-anchoring properties. Protein and lipid are in stick representation. Oxygen and nitrogen atoms are red and blue, respectively. Carbon atoms are gray-colored for wtGLTP, yellow-colored for D48V and orange-colored for a potential alternative conformation around Cβ-Cγ bond. Dashed lines and orange zig-zag line indicate H-bonds and van-der-Waals contact, respectively.

Remark. It is noteworthy that in another crystal form of D48V•diSF (form #1, Table 1), the 6-O-sulfogroup adopts the conformation-2 (Figure 4b) and protein rearranges only the side chain of Tyr207 (Figure S2f). The C-end is still involved in the network of interactions supporting His140, which therefore conserves the usual H-bond with the ceramide amide group. Both sulfo-groups and protein regions under discussion are clearly visible in electron density maps (Figures S2) except for the Tyr207 side chain, which is partially disordered in crystal form #1.

Supplementary Tables

Table S1. Unit cell parameters of 'isomourphous' C2-crystals of GLTP-GSL complexes

GLTP	Ligand	Number of Complexes	Unit Cell		
			**a** (Å)	**b** (Å)	**c** (Å)
WT	Neutral GSLs	5	75.7 (±0.2)	49.2 (±0.1)	68.5 (±0.1)
WT	sulfatides	3	74.8 (±0.2)	50.2 (±0.2)	65.6 (±0.2)
D48V	sulfatides	3	78.0 (±0.1)	48.2 (±0.9)	63.0 (±0.8)
A47D‖D48V	sulfatides	2	76.6 (±0.3)	48.6 (±0.5)	68.5 (±0.5)

Table S2. DLS characterization of lipid- induced protein dimerization.

	Sample name	R(nm)[a]	MW (kDa)[b]	Oligomeric form
**Apoform**	Wild Type	2.2	21	monomer
**Complex**	WT-12:0 monoSF	3.1	46	dimer
**Apoform**	Wild Type	2.3	23	monomer
**Complex**	WT-12:0 diSF	3.1	49	dimer
**Apoform**	D48V mutant	2.2	21	monomer
**Complex**	D48V-12:0 monoSF	3.1	48	dimer
**Apoform**	A47D‖D48V	2.4	26	monomer
**Complex**	A47D‖D48V-12:0 monoSF	2.8	38	dimer

[a] Hydrodynamic radius

[b]Molecular weight estimated from R

Table S3. Number of 'close' monomer•monomer contacts in different crystals

		Number of dimeric contacts[1] in structural elements involved in					
**Ligand**	**Cryst. form**	**α6•α6'**	**α2•α2'**	**$\alpha2_{tip}$•α6'**	**$\alpha2_{tip}$•Cend'**	**Lip•Lip'**	**Lip•Prot'**
	**WT-GLTP**						
12:0 monoSF	#1	25	0	2	2	6	40
	#2	20	0	2	6	2	24
	#3AB	23	0	1	0	6	26
	#3DE	21	0	1	0	13	37
24:1 SF	#1	24	0	0	2	6	24
24:1Gal	#1	23	1	2	2	2	26
12:0 diSF	#1	19	0	0	0	6	34
	#2	11	0	2	6	10	43
	**D48V-GLTP**						
12:0 SF	#1	16	26	4	18	3	18
12:0 diSF	#1	10	18	6	12	10	50
	#4	8	14	4	12	5	56
24:1 SF	#1	12	22	6	14	0	16
	**A47D||D48V-GLTP**						
12:0SF	#1	8	0	4	0	4	40
24:1SF	#1	14	0	4	4	4	20

[1]Only close contacts considered, with the contact distance less than 4 Å
